# Biomarkers in autoimmune diseases of the central nervous system

**DOI:** 10.3389/fimmu.2023.1111719

**Published:** 2023-04-05

**Authors:** Fenghe Zhang, Xue Gao, Jia Liu, Chao Zhang

**Affiliations:** ^1^ Department of Neurology and Institute of Neuroimmunology, Tianjin Medical University General Hospital, Tianjin, China; ^2^ Centers of Neuroimmunology and Neurological Diseases, China National Clinical Research Center for Neurological Diseases, Beijing Tiantan Hospital, Capital Medical University, Beijing, China

**Keywords:** autoimmune diseases, biomarkers, neurofilament light chain, glial fibrillary acidic protein, Kappa free light chain, cytokines, CNS

## Abstract

The autoimmune diseases of the central nervous system (CNS) represent individual heterogeneity with different disease entities. Although clinical and imaging features make it possible to characterize larger patient cohorts, they may not provide sufficient evidence to detect disease activity and response to disease modifying drugs. Biomarkers are becoming a powerful tool due to their objectivity and easy access. Biomarkers may indicate various aspects of biological processes in healthy and/or pathological states, or as a response to drug therapy. According to the clinical features described, biomarkers are usually classified into predictive, diagnostic, monitoring and safety biomarkers. Some nerve injury markers, humoral markers, cytokines and immune cells in serum or cerebrospinal fluid have potential roles in disease severity and prognosis in autoimmune diseases occurring in the CNS, which provides a promising approach for clinicians to early intervention and prevention of future disability. Therefore, this review mainly summarizes the potential biomarkers indicated in autoimmune disorders of the CNS.

## Introduction

With the deep investigation on the pathogenic mechanisms of the CNS, the study of biomarkers has become a particularly active research field because of its potential application in clinical practice in disease diagnosis and prognosis evaluation. They are easy to quantify and can well characterize the autoimmune diseases. Molecular biomarkers combined with imaging tools largely contribute to the diagnosis, evaluation of the efficacy of disease modifying drugs (DMDs), and prediction of disability in clinical practice. At present, Biomarkers with clinical significance and prospects from blood and cerebrospinal fluid (CSF) have been proposed one after another. Therefore, this review collates the biomarkers related to CNS autoimmune diseases and potential clinical significance.

## Neurofilament light chain (NfL)

Neurofilament is the cytoskeleton component of neurons, which is especially rich in axons and plays a structural role in maintaining axon morphology. Neurofilament consists of three subunits, namely, neurofilament light chain (NfL), neurofilament medium chain and neurofilament heavy chain. NfL refers to the neurofilament chain with molecular weight less than 68 kDa, which is the most widely studied component of neurofilament. When axonal or neuronal damage occurs, NfL could be released and can be detected in the CSF and blood (1). Previous studies have found that CSF NfL is associated with disease disability, disease activity, and the time since the last relapse in patients with relapsing and remitting multiple sclerosis (RRMS) (1–7). However, the previous study could only accurately quantify NfL in CSF samples, because the sensitivity of the detection technique is not accurate enough to quantify the level of serum NfL (sNfL) (1). Because lumbar puncture is an invasive operation, it is more suitable for clinical diagnosis and treatment for the study of sNfL. At present, single molecular array (SIMOA) is generally used to determine NfL. Current studies have shown that there is a close correlation between sNfL levels and multiple sclerosis (MS). SNfL is related to disease activity, treatment response, disease and disability progression, and can be combined with magnetic resonance imaging (MRI) to assist in disease diagnosis and treatment (**Table 1**). However, NfL can be increased in any status that leads to axonal damage, including normal aging. Therefore, despite its high sensitivity, it is not an ideal biomarker for a single diagnostic test (8).

As a biomarker of disease activity, NfL can be used to provide a quick overview of disease activity. A large nested case-control study on patients with MS found that the elevation of sNfL was usually several years earlier than the clinical onset of MS. The level of sNfL increased close to the clinical attack and the onset itself was related to the significant increase of sNfL. The inherent increase of sNfL level before symptoms was related to the high risk of MS, suggesting that MS may have a prodromal period lasting for several years, during which axonal damage has occurred (9). Another study showed that patients with higher sNfL levels had a higher risk of relapse than patients with lower levels (10). In addition, sNfL also has a prognostic value in patients with clinical isolation syndrome (CIS) in the conversion to clinically defined MS (10).

As a marker of subsequent attacks, sNfL can also be used as an index to monitor treatment response. It was found that DMD treatment was an independent factor related to sNfL levels. The sNfL level of patients treated with DMD was lower compared with untreated patients, and sNfL level decreased significantly during follow-ups (11, 12). Compared with other treatments, high-efficiency therapies may lead to a greater decline in sNfL levels over time (10). After excluding the influence of confounding factors such as age and body mass index on sNfL measurement, Benkert and his colleagues established a reference database by using the statistical methods of percentile and Z score (13). The results showed that sNfL can be used as a biomarker to predict the individual therapeutic effect and course of MS. In addition, sNfL could be used as an additional measure of disease activity and sNfL concentration could also be used to quantitatively compare the long-term effectiveness of disease modification therapy (DMT) (13). This study showed that in all patients with MS and patients with no evidence of disease activity, a sNfL Z score greater than 1.5 was associated with an increased risk of future clinical or MRI disease activity. And Z score was more accurate compared to the absolute value of sNfL (13).

In addition, the level of sNfL was independently correlated with the score of Expanded Disability Status Score (EDSS) (14, 15), and could be used as a predictor of the long-term course of disability in MS. Several studies found that baseline sNfL levels were significantly correlated with EDSS scores, MS clinical phenotype and treatment response (10). Another study also indicated that as baseline sNfL levels increased (equal to or higher 7.3 pg/ml), the risk of disability progression as measured by relapse-free EDSS-progression also increased, and patients with lower sNfL levels than 7.3 pg/ml were significantly less likely to experience disability progression (16). Patients with secondary progressive MS (SPMS) transition were more likely to show higher sNfL levels during follow-up compared to baseline. The results showed that sNfL measurement could predict disability progress and could distinguish SPMS patients, thus promoting the early diagnosis of patients at risk (16). In addition, some studies have also found that sNfL was related to the EDSS score and seizure severity of myelin oligodendrocyte glycoprotein-IgG associated disease (MOGAD), and may predict the long-term prognosis of MOGAD (17). In a prospective study, the relationship between sNfL levels and disease severity and prognostic indicators of neuromyelitis optica spectrum disorders (NMOSD) was evaluated. The results also showed that sNfL level was positively correlated with EDSS score. Therefore, sNfL may be a biomarker of disease activity and disability in MS, MOGAD and NMOSD (18).

The increase of sNfL level is also related to the loss of brain and cervical spinal cord volume in MS patients (15), and the baseline sNfL level is a predictor of brain atrophy (19). A large observation cohort with a 12-year follow-up in a single center showed that sNfL level was correlated with brain atrophy (10). In a recent study, baseline sNfL predicted brain atrophy in the following 12 and 24 months, with the latter baring the stronger correlation (19). In a group of newly diagnosed CIS and RRMS patients, it was found that the sNfL level at the diagnosis time point was significantly correlated with the baseline T2 lesion volume (20). Besides, a higher baseline sNfL level can predict brain atrophy in the next 2 years (20). After 2 years, the brain volume of patients with higher baseline sNfL decreased faster, and the volume of T2 lesions increased faster (20). Furthermore, higher CSF and sNfL levels in MS patients are associated with more severe gray matter atrophy (21, 22), and CSF NfL concentration is an independent predictor of gray matter volume in CIS (22).

High NfL concentration in serum and CSF is related to Gd enhancement and the number of new/enlarged lesions on MRI, which is not limited to RRMS, but also in patients with progressive MS (8, 15). sNfL level was positively correlated with the existence and number of Gd+ lesions which indicating acute inflammatory neuron injury (20). Within 3 months after Gd+ injury, sNfL level increased (23). In addition, sNfL seems to have the potential to distinguish clinical recurrence with Gd+ lesions from clinical recurrence without Gd+ lesions (23). Therefore, sNfL measurement can be used as a tool in clinical practice to decide when a patient needs MRI enhancement evaluation (20).

## Glial fibrillary acid protein (GFAP)

GFAP is the main intermediate filament that makes up the cytoskeleton of astrocytes, which maintains the integrity of cell structure. It also plays a role in cell mitosis, astrocyte-neuron communication and glial scar formation. The detection of GFAP in CSF or serum reflects the damage of astrocytes (24, 25). There is a close correlation between CSF and serum GFAP (sGFAP) levels in patients with demyelinating diseases (26, 27).

Recently, a large number of studies have investigated the potential of GFAP as an indicator of disability progression, diagnosis and early disease activity in MS (24). One study established a positive correlation between sGFAP and EDSS score or recent attacks, but no therapeutic effect was detected in RRMS. In RRMS, sGFAP levels were associated with the maximum EDSS score indicating disease progression during the most recent attack, but not with the remission between recent attack. In addition, the study analyzed sera samples from 32 RRMS patients in remission and found that the GFAP levels were not higher compared to previous studies. The value of sGFAP to predict subsequent attack in MS warrants further study (26). In addition, CSF GFAP is more likely to be related to progressive MS compared to RRMS (28). Therefore, GFAP may be not sufficient to be an appropriate biomarker for MS diagnosis and disease activity assessment alone, and more biomarkers and imaging evidence are needed to distinguish MS from other neurological diseases.

It was found that in patients with aquaporin-4 (AQP4)-IgG+ NMOSD, sGFAP was also significantly correlated with clinical disability parameters. sGFAP has a potential role as a biomarker of disease severity and future disease activity in patients with AQP4-IgG+NMOSD in clinical remission (29). In addition, sGFAP is not only correlated with disease activity, but also with inebilizumab-treatment response in NMOSD (30). In contrast, no association between sGFAP and clinical disability parameters was observed in patients with MOGAD (29). However, the correlation between sNfL and clinical disability parameters and future disease activity in AQP4-IgG+ patients is to be determined. The potential correlation between sGFAP as a biomarker of disease severity and prognosis of AQP4-IgG+NMOSD deserves further study in an independent cohort of AQP4-IgG+NMOSD patients (29). Another study found a strong correlation between sGFAP levels and EDSS scores and recent attack rates, suggesting that sGFAP levels are biomarkers of disability and disease activity in NMOSD, regardless of age or sex (26). The level of sNfL in NMOSD was higher in patients with higher EDSS score and older patients. sGFAP is associated with recent myelitis attack, but not with clinical disease activity from other anatomical attacks. The results showed that sGFAP and sNfL may be good biomarkers of disease activity and disability, while sGFAP/sNfL quotient at attacks may be a potential diagnostic marker for NMOSD. Higher sGFAP/SNfL quotient at attacks has a sensitivity of 73.0% and specificity of 75.8% to distinguish NMOSD from MS (26). When the disease relapsed, the level of sNfL in NMOSD and MS groups decreased with time, but the decline rate in NMOSD group was slower compared with MS patients. It seems that not sNfL but the combination of sGFAP and sNfL helps distinguish NMOSD from MS (25).

SNfL and sGFAP can also be used as biomarkers of therapeutic effect in NMOSD (31). A recent study showed, tocilizumab and rituximab (RTX) significantly decreased the levels of sNfL and sGFAP at the end of follow-up compared to corticosteroids (32).

## Contactin-1 (CNTN-1)

Contact proteins are a group of cell adhesion molecules, which are mainly expressed in the brain and are indispensable in axon domain organization, axon orientation, neurogenesis, neuron development, synaptic formation and plasticity, axon-glial cell interaction and nerve regeneration. At present, enzyme-linked immunosorbent assays (ELISAs) is generally used to detect the concentration of CNTN-1 in CSF. A previous study showed that the decrease of CSF CNTN-1 level in patients with MS was associated with disease progression, suggesting that CNTN-1 can be used as a new marker of axonal injury (33). Compared with healthy controls, the levels of CNTN-1 and CNTN-2 in RRMS and SPMS decreased at most by 1.4 times, while in patients with CIS, CNTN-1 tended to increase compared with controls. Baseline CNTN-2 levels also play a vital role in predicting longitudinal decline in cortical volume. CSF CNTN-1 levels in SPMS patients were positively correlated with MRI standardized brain volume, but negatively correlated with T2 lesion load. As for RRMS and primary progressive multiple sclerosis (PPMS), there was no correlation between CNTN-1 and CNTN-2 and standardized brain volume or T2 lesion load (34). A recent study has shown that serum CNTN-1 (sCNTN-1) can be used as a biomarker of long-term disease progression in MS. According to a 3-year prospective study, median sCNTN-1 levels were significantly lower in RRMS patients with natalizumab-treated compared with healthy controls. It also found that sCNTN-1 levels in RRMS patients with disability progression decreased significantly before and 12 months after treatment compared with non-progressive patients (35). Therefore, CNTN-1 can be used as a sensitive biomarker of disease activity and also as a biomarker of therapeutic response (**Table 1**). It can complement MRI and clinical evaluation in the process of diagnosis, but more studies are needed to verify the pathogenesis of CNTN-1 and its role in MS pathology.

## Chitinase-3-like protein 1 (CHI3L1)

Chitinase3-like protein1 (CHI3L1), a secretory glycoprotein, is one of the newly discovered markers of inflammation in recent years, which can mediate inflammation, macrophage polarization, apoptosis and carcinogenesis. But its physiological and pathophysiological role in the development of cancer and neurodegenerative diseases is still unclear. In human, CHI3L1 is also called chitin protein-40 (chitinaseprotein-40, YKL-40), based on its three N-terminal amino acids, tyrosine (Y), lysine (K) and leucine (L) (36). The relative molecular weight is about 40 kDa. It is a chitin-binding lectin and belongs to the glycosyl hydrolase family 18 (36). In CNS disorders, CHI3L1 is expressed in astrocytes and microglia/macrophages, mainly in active demyelinating areas. Levels of CHI3L1 in the CSF were reported to be increased during acute inflammation of demyelinating disease (37). Patients with SPMS and PPMS had significantly higher levels of CHI3L1 compared to RRMS and CIS in CSF and blood samples. Patients with RRMS were more likely to show high NfL with low CHI3L1 levels (38, 39). However, the expression of CHI3L1 in peripheral blood was affected by many factors, and the specificity for CNS disorders was lower compared to CHI3L1 in CSF. A number of studies have shown that CHI3L1 in CSF is helpful to distinguish the progressive MS and RRMS (38–40). The elevated level of CHI3L1 is a characteristic of progressive disease. In patients with RRMS, high level of CHI3L1 in CSF is an independent predictor of the deterioration of neurological dysfunction and the progression of the disease to SPMS. Therefore, CHI3L1 in CSF may predict the progression of RRMS (37–40). In addition, CHI3L1 has a good prognostic effect in the early MS and has the potential to become a therapeutic target in MS (38, 41).

## Kappa free light chain (KFLC)

Kappa chain (κ chain) is a kind of Ig molecular light chain (L chain), which is mainly produced in the sheath in the CNS. The light chain of Ig includes Kappa chain (κ chain) and Lambda chain (λ chain) (42). FLC in serum is mainly cleared by kidney, but this process does not exist in CSF. Thus quantitative detection of FLC combined with blood-brain barrier function can reflect the synthesis of intrathecal Ig, which can be used in the diagnosis and prognosis of CSF inflammatory and infectious diseases (43). Electrophoresis is generally used to detect KLFC in CSF. Compared with oligoclonal band (OCB), CSF KFLC has higher sensitivity in the diagnosis of MS/CIS, and there is no significant loss of specificity. Studies have shown that the determination of CSF KFLC is a valuable quantitative substitute or supplement for the qualitative evaluation of OCB (43, 44). The total amount of intrathecal KFLC synthesis can distinguish MS myelitis from NMOSD myelitis. KFLC IF (intrathecal fraction) > 78% can distinguish myelitis caused by MS and NMOSD, with a sensitivity of 88.5% and a specificity of 88.9% (45). In addition, KFLC has high stability and has additional advantages over OCB, such as objectivity, easier standardization, faster speed, lower cost and so on (43, 44). However, steroids have a significant effect on KFLC levels (46), and further studies are needed to determine how much steroid treatment affects KFLC levels.

## KFLC index

KFLC index is a method for measuring the production of KFLC in the sheath. The index is obtained by linear modeling to calculate the concentration of KFLC in serum and CSF. The formula of KFLC index is: FLC index = Q _FLC/_Q _alb_ with Q _FLC_ = CSF FLC/serum FLC and Q _alb_ = CSF albumin/serum albumin (47). Similar to CSF KFLC, KFLC index is better than OCB in the diagnosis of MS and differentiation of MS and other inflammatory CNS disorders (44, 47, 48), so KFLC index may replace OCB as a first-line biomarker of MS in clinical practice. The early initiation of DMDs in MS is important to slow down progression in disability and cognitive impairment. KFLC index predicted the second clinical attack in patients with CIS in both space and time (47), and high KFLC index was an independent risk factor for early further attacks. In a prospective cohort study, a 10% increase in the KFLC index indicates an increase in the risk of a second clinical attack of about 13%. Patients with a high KFLC index (> 100) are twice as likely to have a second clinical attack within 12 months as those with a low KFLC index (49). Compared to OCB, KFLC index has methodological advantages in the diagnosis of MS and is independent of subjective interpretation (48). KFLC index may be not affected by DMT, demographic factors, clinical demyelination event types or MS phenotypes (47, 48). Young age, female sex and evidence of disease activity are independent factors associated with high KFLC index of MS (47). Current evidence suggests that the KFLC index is a reliable prognostic biomarker that may replace the OCB assay and bring us closer to the tailored drugs of MS.

## Anti-AQP4-Antibody (AQP4-IgG)

AQP4 is a widely expressed water channel mainly expressed in astrocytes of the CNS, especially in astrocytes involved in the formation of the blood-brain barrier (50). AQP4-IgG positive NMOSD is marked by the destruction of astrocytes (51). Current studies have shown that clinical, pathological and preclinical evidence consistently support the pathogenic role of AQP4-IgG in NMOSD. AQP4-IgG exists in up to 70% ~ 90% of NMOSD patients and is highly specific for the disease (52, 53).The titer of AQP4-IgG in serum is more than 500 times higher than that in CSF, so only patients with negative serum should be considered for CSF detection to improve sensitivity. The detection of AQP4-IgG in serum is necessary in the diagnostic criteria of the international expert group of NMOSD in 2015 (52). The laboratory method with the highest sensitivity and specificity for detecting AQP4-IgG is cell-based array (CBA) detection (54), with a sensitivity and specificity of 76% and 99%, respectively. However, limited evidence indicate that AQP4-IgG serum status cannot be used as a biomarker to predict disease activity and immunosuppressive drug response (52).

## Anti-MOG-Antibody (MOG-IgG)

Myelin oligodendrocyte glycoprotein (MOG) is usually found on the surface of mature oligodendrocyte and the myelin sheath of the CNS, and its expression begins in the late stage of myelin formation (53). It may play a structural role in microtubule stability, myelin fiber adhesion and response to inflammation. MOG-IgG usually belongs to IgG1 subclass. It can activate the complement cascade and determine the disorder of the cytoskeleton of oligodendrocytes, resulting in demyelination (52). At present, the best method to detect MOG-IgG is CBA. In the past 40 years, the pathogenicity of autoimmune response to MOG has been well confirmed (55). Acute disseminated encephalomyelitis (ADEM) is the most common clinical manifestation associated with anti-MOG antibodies. Anti-MOG antibody was only briefly observed in monophasic diseases such as ADEM, and its decrease was associated with a good prognosis, but persisted in polyphasic ADEM, NMOSD, relapsing optic neuritis or myelitis (56). The titer of MOG-IgG fluctuates during the clinical course of the disease and the level is higher in the acute attacks (57). However, the titer of MOG-IgG was not related to the risk of relapses or the final clinical outcome (58). In early studies, the lack of disease specificity was revealed by testing MOG-IgG at low titers in MS patients or other neurological diseases or even in healthy individuals (59). These observations suggest that MOG-IgG in the serum may bind to MOG and produce non-specific positive signals, or these antibodies may belong to the natural antibody class that is relatively common at low levels and will not be deleted by the B cell tolerance mechanism, but do not cause disease (59). Some studies have shown that MOG-IgG is associated with AQP4-IgG seronegative NMOSD. Compared with AQP4-IgG seropositive patients, MOG-IgG seropositive patients have a lower risk of further relapses and a better visual field prognosis (54). MOG-IgG is associated with clinical manifestations and younger age of onset of human inflammatory demyelinating diseases, with the highest incidence in pediatric patients (56, 59). The available evidence shows that MOG-IgG can be used as a prognostic biomarker of MOGAD (54).

## Autoimmune encephalitis associated antibody

Autoimmune encephalitis (AE) is characterized by the existence of autoantibodies that directly attack the protein in or on the surface of neurons, so the detection of protein-specific antibodies in CNS has changed our understanding of AE and our ability to make an accurate diagnosis (60). At present, the best method to detect these antibodies is CBA. These antibodies target important brain proteins, including neurotransmitter receptors, ion channels and related membrane proteins (60). They are specific for a definite diagonis of AE. For example, the only specific diagnostic test against N-methyl D-aspartate receptor (NMDAR) encephalitis is to prove the IgG autoantibodies targeting the GluN1 subunit of the NMDAR in CSF of patients (61). The antibody of anti-LGI1 encephalitis is IgG antibody targeting leucine-rich glioma-inactivated 1 (LGI1) protein (an extracellular component of the voltage-gated Kv1 potassium channel-complex) (62). This study has confirmed that IgG4 is the major subclass of LGI1-IgG, and a higher LGI1-IgG specific CSF index, that is, the index of intrathecal antibody synthesis, is related to the poor prognosis of patients with anti-LGI1 encephalitis (62). Autoimmune GFAP astrocytopathy is a meningoencephalitis associated with GFAP-specific IgG (63). GFAP-specific IgG can be used as a biomarker of recurrent autoimmune meningoencephalitis that responds to immunotherapy (64). Positive sGFAP specific IgG can distinguish autoimmune GFAP meningoencephalitis from other diseases (64). One of the types of AE that is difficult to diagnose is antibody-negative AE, because there is no definite antibody or known AE syndrome to explain this manifestation (65). In a cohort of children, antibody-negative AE was associated with poor cognitive outcomes compared with NMDAR encephalitis (66). The diagnosis of AE without antibody recognition is usually made without alternative diagnosis, such as neuroimaging, CSF analysis and electroencephalogram (EEG) (65). Recognition of the characteristic examinations in the limbic system of AE is an important clue to guide the diagnosis (67). Brain MRI may be normal, non-specific, or show multifocal T2/FLAIR high signal changes (68). EEG has no specific pattern association with most AE subtypes, except for the extreme delta brush pattern found in NMDAR encephalitis (69). EEG may help to distinguish organic and mental pathology (70). EEG may also serve as a biomarker of disease severity to guide treatment decisionmaking (70). CSF detection is very important because classical CSF analysis provides more timely information, such as CSF leukocyte count, total protein and OCB of CSF, which may support the diagnosis of AE (71). The presence of specific CSF and serum autoantibodies is extremely important for the final diagnosis of AE. CSF antibody detection is more sensitive and specific, so it is better than serum antibody detection (72).

## Cytokines

Cytokines and chemokines have multiple effects on many inflammatory cells, most of which have unique characteristics and are elevated in many neuroimmune diseases of the CNS. Therefore, cytokines and chemokines can be used as biomarkers for diagnosis of autoimmune disease and detection of intrathecal inflammation, which can be used to evaluate disease activity and to predict disease progression (73). Chemokine is a secretory protein that controls the transport and localization of leukocytes to the target organ (74). At present, ELISA is generally used to detect these biomarkers. Soluble inflammatory mediators have long been studied as appropriate biomarkers that can predict the process of MS (75). Some of these soluble markers are not disease specific, and the challenge of biomarker research is still lack of repeatability and sensitivity. However, some candidate biomarkers have been studied and need to be verified, and” omics “technology is developing rapidly, providing a basis for future research (76).

### IL-6

Interleukin-6 (IL-6) is considered to be an important cytokine in inflammatory diseases of the CNS. It has multiple functions and mediates many biological activities. It participates in acute inflammation by inducing the synthesis of acute phase proteins, so the increased concentration of IL-6 in CSF may represent a non-specific marker of inflammation in the CNS (77). In addition, IL-6 is also one of the B cell stimulating factors, which differentiate B cells into plasma cells and lead to the production of immunoglobulin (78). IL-6 is significantly increased in serum and CSF of NMOSD patients, which may play a variety of roles in the pathophysiology of NMOSD by promoting plasma cell survival, stimulating the production of anti-AQP4 antibody, destroying the integrity and function of blood-brain barrier and enhancing the differentiation and activation of pro-inflammatory T lymphocytes (79). Blocking IL-6 signal transduction with anti-IL-6 receptor monoclonal antibody tocilizumab is very effective for refractory NMOSD patients (80). The increased CSF IL-6 level at diagnosis is associated with increased recurrence and disability in RRMS patients during the 3-year follow-up (77). In addition, the ratio of IL2:IL6 in CSF may be a prognostic biomarker of early MS and may be helpful to predict the early relapsing activity of MS (81). CSF IL-6 can also be used as a biomarker to distinguish NMO from MS (82). Besides, the increase of serum IL-6 level in AE patients may indicate the persistent proinflammatory state of AE and may lead to poor prognosis (83). In anti-NMDAR encephalitis, with the increase of cytokines including IL-6, the clinical symptoms are aggravated (84). Therefore, IL-6 may be a new biomarker of anti-NMDAR encephalitis.

### IL-17A

Interleukin-17A (IL-17A) is an effective proinflammatory cytokine produced by Th17 cells and IL-17-secreting CD8+T cells (85). It promotes the pathophysiology of autoimmune diseases and may mediate delayed inflammation by inducing neutrophils and monocytes to recruit chemokines at inflammatory sites (85). A recent study has shown that IL-17 impairs myelin regeneration and promotes myelin damage through oligodendrocyte/myelin injury mediated by increasing voltage-gated K+ channel 1 (86). Some researchers speculate that the level of IL-17A may increase in the early stage of MS inflammation, and the level of IL-17A will gradually decrease with the remission of inflammation, but this speculation still needs further study (87). IL-17A may regulate inflammatory immune response in NMOSD through PI3K-m TOR signaling pathway, and promote disease progression (88). The high expression of IL-17A in peripheral blood of patients with relapsing NMOSD suggests that it may be related to relapse (88). The concentration of pro-inflammatory IL-17A in CSF of AE patients increased and correlated with the severity of the disease at the time of onset (89). Therefore, IL-17A in CSF can be used to evaluate the short-term severity of AE patients and can lead to early immunosuppressive therapy (89).

### CXCL13

Chemokine (C-X-C motif) ligand 13 (CXCL13) is an effective B cell chemical attractor, which is essential for B cell migration and the development of B cell follicles and secondary lymphoid structures (73). CXCL13 is increased in patients with autoimmune diseases and is related to the disease severity, activity and prognosis (90). A meta-analysis shows that CSF CXCL13 and blood IL-23 levels in patients with MS are always different from those in healthy controls, and they may be used for diagnostic purposes (91). Increased concentration of CXCL13 was detected in blood, CSF and active demyelinating brain lesions in patients with MS (90). Notably, in patients with RRMS, CSF CXCL13 levels were associated with increased relapsing rates and disease severity measured by the EDSS (90). The level of CSF CXCL13 in MS patients decreased significantly after treatment (91). Therefore, CSF CXCL13 may be used in the diagnosis of MS in clinical practice, and may also become a biomarker of drug treatment response and disease progression of MS (91). Further research is needed to verify this. Besides, CXCL13 may also be a promising biomarker for the course of AE (82). For example, it has been shown that the increase of CSF CXCL13 in 70% of patients with early anti-NMDAR encephalitis is associated with intrathecal NMDAR antibody synthesis (82).

### Osteopontin

Osteopontin (OPN), also known as secreted phosphoprotein-1 (SPP1), is mainly released by endothelial cells, microglia, macrophages and dendritic cells in the brain (92). OPN mainly reflects the activation of innate immune system and promotes inflammation by increasing the production of IL-12, IL-17 and interferon-γ (IFN-γ) and inhibiting the expression of IL-10 (93). OPN tends to induce proinflammatory cytokines in NMOSD and MS (94). Plasma OPN levels in patients with NMOSD were higher than healthy controls, especially in the cases with attacks and severe disability (95). High levels of OPN can be detected in CSF, serum or plasma in patients with MS, indicating that the protein may be used as a biomarker for monitoring disease activity and progression (95). A meta-analysis showed that OPN levels in CSF and blood in patients with MS were significantly elevated, and OPN levels in CSF in active MS patients were significantly compared to non-active patients (93). OPN may play a harmful role in the development of MS (92) and is closely related to disease activity (92, 96). The levels of tumor necrosis factor and OPN in CSF of patients with early RRMS after dimethyl fumarate treatment are related to disease activity (96). Therefore, the combined detection with OPN and other markers can help clinicians make personalized treatment strategies. Moreover, OPN in CSF can predict the development of lesions and microstructural abnormalities within 10 years (97). The increase of OPN concentration in CSF indicates the enlargement of lateral and inferior ventricles in progressive MS, accompanied by changes in cortical and subcortical gray matter and white matter volume (94). Higher OPN levels in CSF indicate poor prognosis and long-term disease progression and are associated with the deterioration of the disease (94, 98). More and more evidence shows that OPN can be used as a biomarker for clinical diagnosis or prediction and prognosis.

### Combined detection of cytokines

The combined detection of these cytokines may be more helpful to the prediction and evaluation of diseases. Moreover, CSF CXCL13, CXCL8 and IL-12p40 can be used as biomarkers to predict the progression from CIS to MS (74). Increased concentrations of IL-6, IL-17 and CXCL13 are considered to be key factors in inducing the formation of NMO lesions (82). These molecules have been shown to be associated with the severity of NMO disease and EDSS scores (82). IL6, IL-17A, CXCL10 and CXCL13 in CSF can be used to detect inflammation in acute stage of AE (99). The elevated levels of cytokines such as CXCL-13, CXCL-10, IL-6 and IL-17A are related to the clinical severity of anti-NMDAR encephalitis (100). The levels of IFN- γ, IL-17, IL-12 and IL-23 in the CSF of AE patients with positive autoantibodies to cell surface protein are higher than those of AE patients with positive autoantibodies to intracellular antigens (101).

## Cell markers

### Memory B cells and plasma cells

The antigen presentation process not only activates autoreactive T cells, but also induces the proliferation of presenting B cells and their subsequent differentiation into memory B cells and antibody-producing plasma cells (102). At present, flow cytomety is generally used to detect cell markers. Memory B cells (CD19+/CD27+), as part of the secondary immune response, can rapidly produce immunoglobulins. Many studies indicate that CD19+/CD27+ memory B cells can be used as biomarkers for RTX treatment monitoring and retreatment in patients with NMOSD. Class-switched memory B-cells (CD19+/CD27+/IgM-/IgD-, SMB), an early regenerated memory B cell subset, may also be a sensitive biomarker of disease recurrence risk (103). By monitoring the memory B cells or SMB cells in peripheral blood mononuclear cells, individualized RTX administration regimens for NMOSD patients can be made without losing the efficacy while reducing the cumulative dose and medical expenses of RTX (103–105). Plasma cells (PCs) represent the terminal differentiation from mature B cells and play a key role in effective short- and long-term humoral immunity by producing a large number of antigen-specific antibodies (106). A recent study shows that CSF plasmablasts can distinguish MS from other neurological diseases (107).

### Eomes+ Th cells

Recent studies found that cytotoxic CD4+T cells expressing Eomes (Eomes+ Th cells) may play an important role in the pathogenesis of SPMS and have the potential value of distinguishing biomarkers between SPMS and RRMS patients (**Table 1**) (108). Eomes+ Th cells from experimental autoimmune encephalomyelitis lesions and the blood of SPMS patients can release cytotoxic granzyme B and IFN- γ and up-regulate CD107a (also known as lysosomal associated membrane protein 1) (109, 110). Granzyme B released by Eomes+ Th cells binds and activates protease-activated receptor-1 on the surface of neurons and leads to neurodegeneration (109). Compared with healthy subjects and RRMS patients, Eomes+ Th cells in peripheral blood and CSF were significantly increased in SPMS patients (109). The detection of Eomes+ Th cells is of great value for SPMS diagnosis and prognosis monitoring. The accuracy of Eomes+ Th cell level as a biomarker to predict the risk of disease progression in SPMS patients was more than 80% (108). In addition, Eomes+ Th cells may also be a potential therapeutic target for SPMS patients (109).

**Table 1:** Overview of the biomarkers described in this review.

Biomarkers	Common Related Diseases	Function
CNS Injury Markers		
NfL	MS, NMOSD	To monitor disease activity, To monitor drug treatment response, To predict disability progression, To aid imaging diagnosis
GFAP	NMOSD	To monitor disease activity and disability progression, To monitor drug treatment response, To aid differential diagnosis
CNTN-1	MS	To monitor disease activity, To monitor drug treatment response
CHI3L1	MS	To predict disability progression
Humoral Markers		
KFLC	MS	Diagnostic and prognostic markers, To monitor disease changes and efficacy
KFLC Index	MS	To aid diagnosis and differential diagnosis Prognostic markers
AQP4-IgG	NMOSD	Diagnostic markers
MOG-IgG	MOGAD	Prognostic markers
Autoimmune encephalitis associated antibody	AE	Diagnostic markers
Cytokines		
IL-6	NMOSD, MS	Prognostic markers
IL-17A	AE	To evaluate short-term severity
CXCL13	MS, NMDAR encephalitis	Diagnostic and prognostic markers, To monitor drug treatment response,
OPN	MS	To monitor disease activity and progression, Diagnostic and prognostic markers
Cell Markers		
Memory B cells and plasma cells	NMOSD, MS	To monitor drug treatment response
Eomes+ Th cells	MS	To monitor disease progression, Prognostic markers

CNS, central nervous system; NfL, neurofilament light chain; GFAP, glial fibrillary acid protein; CNTN-1, Contactin-1; CHI3L1, chitinase3-like protein1; KFLC, kappa free light chain; AQP4-IgG, aquaporin-4-IgG; MOG-IgG, myelin oligodendrocyte glycoprotein-IgG; IL-6, interleukin-6; IL-17A, Interleukin-17A; CXCL13, chemokine (C-X-C motif) ligand 13; OPN, osteopontin; MS, multiple sclerosis; NMOSD, neuromyelitis optica spectrum disorders; MOGAD, myelin oligodendrocyte glycoprotein-IgG associated disease; AE, autoimmune encephalitis; NMDAR, N-methyl D-aspartate receptor.

## Conclusion

We have systematically reviewed the potential biomarkers of CNS autoimmune diseases. With the improvement of diagnostic methods, neurologists can make faster and more accurate diagnosis of CNS autoimmune diseases, so as to significantly improve the treatment response of patients and reduce the rate of disability. However, at present, many biomarkers cannot be used as independent markers in the clinical diagnosis and treatment of diseases, so the joint detection of biomarkers can better achieve the purpose of detection. Although the current research on biomarkers of CNS autoimmune diseases is very extensive, it is still not perfect and more in-depth research is needed.

## Author contributions

FZ and CZ contributed to the conception and design of the study. XG, JL, and CZ contributed to the supervision of the manuscript. XG and CZ edited the paper scientifically. All authors contributed to the article and approved the submitted version.
